# Sex differences in verbal fluency: the role of strategies and instructions

**DOI:** 10.1007/s10339-017-0801-1

**Published:** 2017-04-01

**Authors:** Andrea Scheuringer, Ramona Wittig, Belinda Pletzer

**Affiliations:** 1 Department of Psychology, Paris-Lodron University Salzburg, Hellbrunnerstraße 34, 5020 Salzburg, Austria; 2 Center of Neurocognitive Research, Paris-Lodron University Salzburg, Hellbrunnerstraße 34, 5020 Salzburg, Austria

**Keywords:** Verbal fluency, Sex differences, Clustering, Switching, Effect of instruction, Semantic fluency, Phonemic fluency

## Abstract

Sex differences in verbal fluency performance and strategies are highly controversial, nevertheless suggesting a slight female advantage at least for phonemic fluency. A tendency of increased clustering of words into phonemic and semantic subcategories in men and increased switching between those categories in women has been suggested. In spatial tasks, it has been demonstrated that changes in instructions favoring a certain cognitive strategy can alter sex differences in performance. Such an approach has, however, not been attempted previously with verbal tasks. In the present investigation, 19 women in their luteal cycle phase and 23 men performed a phonemic and a semantic fluency task with three different instructions, one neutral, one emphasizing the clustering, and one emphasizing the switching of words. While under neutral instructions no sex differences were observed in verbal fluency performance and strategies, sex differences in switching and overall performance were observed in semantic fluency with an instruction requiring a switching strategy. Furthermore, correlation analyses suggested that the importance of strategies for overall performance differed between women and men. While only switching, but not clustering was related to overall verbal fluency performance in all tasks under all instructions, this relationship was driven by women in the phonemic task, but by men in the semantic task. These results highlight the importance of a consistent methodology in sex difference research. Slight variations in instructions may in part explain inconsistencies regarding sex differences in verbal fluency between previous studies.

## Introduction

Sex differences in verbal abilities have attracted considerable research interest. While a quite robust and consistent male superiority has been observed in spatial tasks (e.g., Andreano and Cahill 2009; Linn and Peterson 1985; Voyer et al. 1995; Weiss et al. 2003a), a female advantage in verbal abilities is highly controversial (Andreano and Cahill 2009; Hyde and Linn 1988; Kimura 1992).

The perhaps most-cited meta-analytic review, performed by Hyde and Linn (1988), observed that only 27% of the considered studies show a significant female advantage in verbal tasks, whereas 7% of studies found a male advantage. No sex differences were observed in the remaining studies. They concluded that the size of sex differences in verbal abilities is nearly negligible, also considering the small effect sizes (Hyde and Linn 1988). However, one potential problem of large meta-analyses investigating sex differences in verbal abilities may be that they do not differentiate between different categories of verbal abilities. Verbal abilities include a range of skills, e.g., articulation, recall of word definitions, grammar skills, verbal reasoning and language acquisition (Hyde and Linn 1988; Wallentin 2009).

Two domains have been repeatedly considered with respect to possible differences between men and women in the verbal domain, i.e., verbal memory and verbal fluency tasks (Andreano and Cahill 2009; Hyde and Linn 1988; Kimura 1992; Wallentin 2009; Weiss et al. 2003a). In verbal fluency tasks participants have to produce as many words as possible beginning either with the same letter (phonemic fluency) or belonging to the same category (semantic fluency) under time constraints (Troyer et al. 1997).

A female advantage regarding the overall number of words produced has more often been observed in phonemic fluency (Burton et al. 2005, Capitani et al. 1998; Halari et al. 2006; Herlitz et al. 1999; Hyde and Linn 1988; Filippetti and Allegri 2011, Wallentin 2009; Weiss et al. 2003a, 2006), whereas fewer studies indicate that women outperform men in semantic fluency tasks (Capitani et al. 1999, 2005; Filippetti and Allegri 2011; Munro et al. 2012). Some studies even report a male advantage in semantic fluency (Kempler et al. 1998). Nevertheless, even in phonemic fluency, differences between men and women are mostly weak or even missing (Lewin et al. 2001; Tombaugh et al. 1999; Troyer 2000; Weiss et al. 2003b). Tombaugh et al. (1999) for example investigated phonemic and semantic fluency in a large sample of 1300 participants observing absolutely no sex differences. While the influence of sex is highly controversial, an impact of age and education has been observed quite consistently (Capitani et al. 1999: Tombaugh et al. 1999; Troyer et al. 1997; Troyer 2000).

Although both forms of fluency tasks share some internal processing mechanism, phonemic fluency is assumed to be more demanding compared to semantic fluency (Gourovitch et al. 2000; Henry and Crawford 2004; Kavé et al. 2008; Milner and Petrides 1984; Mummery et al. 1996; Troyer et al. 1997;  Paulesu et al. 1997; Unsworth et al. 2010). This is attributed to an increased attention load because the retrieval of words within categories in the semantic task is assumed to be easier than the retrieval of words without categories in the phonemic task. In the semantic task, associations can be built more easily, whereas in the phonemic task the retrieval requires the exploration of more subset categories (Martin et al. 1994; Martins et al. 2007; Troyer et al. 1997; Unsworth et al. 2010).

Two main (solution) strategies are described for verbal fluency (Troyer et al. 1997). First, clustering is characterized as generating consecutive words belonging to the same subcategory, which is described as a relatively automatic process. Switching, the second strategy, is described as consecutive generation of words belonging to different subcategories (Troyer et al. 1997). The switching strategy is considered as requiring more cognitive flexibility and thus refers to a more effortful process than clustering. Furthermore, it is suggested that switching is the more successful strategy for overall verbal fluency performance (Koren et al. 2005; Weiss et al. 2006).

There is some evidence that women switch more often between categories, whereas men generate broader clusters than women (Weiss et al. 2006; Lanting et al. 2009). A large-scale study by Lanting et al. (2009) indicates that sex differences in strategy use during verbal fluency are stable over a wide age range. However, comparable to sex difference in overall fluency performance some investigations failed to demonstrate sex differences in strategy use (Brucki and Rocha 2004; Troyer et al. 1997; Troyer 2000). Again, age but also education showed an impact on switching and clustering in verbal fluency, at least to a small degree (Brucki and Rocha 2004; Sauzéon et al. 2010; Troyer et al. 1997; Troyer 2000). The idea of differential strategy use in men and women is in line with the more consistent observation of sex differences in phonemic tasks, since semantic fluency tasks support the formation of subcategories, i.e., the clustering strategy.

During spatial tasks, it has been demonstrated that the differential strategy use between men and women can in part explain sex differences observed in these tasks. For example, it has been demonstrated that spatial navigation and mental rotation performance are related to the use of a more allocentric strategy use during spatial navigation (Saucier et al. 2002). Likewise, for the verbal fluency task, it has been suggested that a female superiority, if observed, may stem from their stronger tendency to switch between categories during word generation, since switching has been related to better overall performance (Weiss et al. 2006).

Furthermore, it has been demonstrated that performance in spatial tasks can be modulated by the use of different instructions, which in turn affects sex differences in the task. For example, the male superiority in mental rotation and spatial memory disappears, if instructions are changed to not focus on the spatial nature of the task (Sharps et al. 1993). Likewise, a female superiority in spatial navigation can be elicited, if instructions are formulated in egocentric terms (Saucier et al. 2002). Similarly, it was observed for object location memory that different stimulus materials elicited a female superiority if they supported the labeling of stimulus parts and a male superiority if they emphasized the spatial components of the task (e.g., Lewin et al. 2001; Postma et al. 1998). These results suggest that instructions are crucial for sex difference studies and inconsistencies between studies may in part be the result of different formulations used in the instructions. However, the effect of instruction on sex differences and strategy use has not been investigated previously in verbal tasks.

In the present study different instructions, emphasizing either clustering or switching, were combined with a phonemic and a semantic fluency task to investigate whether they affected sex differences in word generation by focusing on clustering of or switching between words. In particular, we hypothesize that men should produce more words than women with the clustering instruction, while women should produce more words than men with the switching instruction.

## Methods

### Participants

 Participants were 42 students from the University of Salzburg, 19 women (*M* = 22.9 years, SE = 0.44 years) and 23 men (*M* = 22.7 years, SE = 0.58 years). Men and women had comparable educational backgrounds (all had finished their A-levels), and age did not differ between men and women (*t*_(39)_ = 0.25, *p* = 0.84).

All participants were right-handers and had no psychological, neurological or endocrinological disorders. Furthermore, only females using no hormonal contraceptives were allowed to participate. Since the strongest sex differences in verbal tasks have been observed during the luteal cycle phase (Hampson 1990), when progesterone and estradiol levels are high, all test sessions for women were scheduled during the luteal cycle phase (3–10 days postovulation). Ovulation was determined by verbal reports from participants of their cycle duration and date of their last period and confirmed by commercial ovulation tests. Ovulation kits test for the LH surge in urine and are available in each drug store. Furthermore, participants informed us about the onset of the next menstruation for the follow-up evaluation of their menstrual cycle phase at the day of testing.

All subjects gave their informed written consent to participate in the study. All methods went conform to the Code of Ethics of the World Medical Association (Declaration of Helsinki).

### Verbal fluency task

#### Procedure

All participants completed hand-written phonemic and semantic verbal fluency tasks. Hand-written versions of the verbal fluency task were previously employed, e.g., by Burton et al. (2005), Herlitz et al. (1999), Lewin et al. (2001) and Weiss et al. (2003a) and were chosen in the present study to allow for more efficient testing in group settings. We used three different instructions for both the phonemic and the semantic fluency task. In total, 19 participants completed first the phonemic and then the semantic task; 22 participants completed first the semantic and then the phonemic task. Following each instruction, participants were given three letters or three semantic categories and had to name as many words as possible starting with that letter or belonging to that category within 1 min. Thus, each participant produced 18 wordlists in total. Letter triplets were PAN, SEM, FOT. Category triplets were Animals-Furniture-Clothing, Fruits-Jobs-Male Names, Vegetable-Toys-Female Names. Letter and category triplets were randomly assigned to the three different instructions.

#### Instructions

Each task started with a neutral instruction, also labeled as baseline condition, during which participants were simply asked to write down as many words as possible starting with the given letter or belonging to the given category without any further restrictions. This varies from the more common instruction insofar as participants were not informed that proper names and variants of the same word (e.g., the same word with different suffixes like orange vs. oranges) would be excluded from the word count (see Troyer et al. 1997). The rationale for deviating from the common practice was to keep the neutral instruction as neutral as possible and not emphasize any strategy. The information that variants of the same word are to be avoided could emphasize a switching strategy. The second and third instruction, which emphasized clustering or switching, was counterbalanced across participants. In total, 19 participants received first the clustering and then the switching instruction during both the phonemic and the semantic task. In total, 23 participants first received the switching and then the clustering instruction during both tasks.

During the clustering instruction participants were asked to make sure that the consecutive words they produced were as phonemically similar as possible in the phonemic task or belonged to the same subcategory in the semantic task. For example, in the phonemic task, the words should rhyme, differ in only one letter, or share the same second letter. In the semantic task, if the main category was “animals,” the first subcategory could be “pets” (cat, dog, rabbit) and another subcategory could be “insects” (bee, butterfly, bug). Participants were given an example of clustering for each task and had to confirm that they understood the construct. Participants were instructed to only switch to another phonemic or semantic cluster, if they could not think of any more words belonging to the same cluster.

During the switching instruction participants were asked to make sure that the consecutive words they produced were as phonemically dissimilar as possible or belonged to a different subcategory in the semantic task. For example, in the phonemic task, the words should not rhyme, differ in more than one letter and share the same second letter. During the semantic task if the main category was “animals,” participants should find animals that were as dissimilar as possible (e.g., sword fish, dog, snake). Participants were given an example of switching for each task and had to confirm that they understood the construct.

#### Scoring

For each wordlist, i.e., each letter or category, the total number of words produced, the average size of clusters, and the number of switches between clusters were evaluated and averaged over the three letters/categories presented with the same instruction. Rules for counting words, cluster size, and the number of switches were used as suggested by Troyer et al. (1997). Errors and word repetitions were excluded from analyses. As we used a written version of the verbal fluency task, homonyms, referred as different spellings of the same word, which were included in the oral version illustrated by of Troyer et al. (1997) could not count as separate words in the present written investigation and thus counted as repetition.

The cluster size was counted starting with the second word in a cluster. To obtain the average cluster size per wordlist, the sizes of all clusters in one wordlist were summed up and divided by the number of clusters in this wordlist.

In the phonemic fluency task, *s*uccessively generated words were taken as a cluster, if the words began with at least the same two letters, such as “fun” and “fund,” if the words rhymed, such as “sand” and “stand,” or if the words differed only in one vowel sound, such as “fun” and “fan.” Words with the same meaning did not count as cluster in the phonemic task, but only in the semantic fluency task. In the semantic fluency task, successively generated words were taken as a cluster if they belonged to the same subcategory, as exemplified for the category “animals” above.

In the case of categories overlapping with some items belonging in both successively following clusters, the two clusters were transformed into one big cluster. For example, “cat, dog, guinea-pig, pigeon, bluebird, duck” is one cluster with the size 5, built of two overlapping clusters (“cat, dog, guinea-pig, pigeon”—one cluster referring to pets, “pigeon, bluebird, duck”—one cluster referring to birds).

Errors and repetitions, which did not count for the overall number of words, were included for the cluster size and the number of switches in both tasks, because these measures should reflect participants’ strategies (see Troyer et al. 1997).

### Analyses

Statistical analyses were performed using software SPSS Statistics 22.0 and will be described in detail in the respective paragraphs of the results section. We used a series of ANOVAs to establish the following:

The first set of analyses (see “Neutral instructions” section) is focused on the neutral condition to assess (1) sex differences in overall performance (as indicated by the total number of words produced), and (2) sex differences in verbal fluency strategies (as indicated by cluster size and number of switches).

The second set of analyses (see “Effect of instruction and sex on overall verbal fluency performance (number of words produced)” section) is focused on the effect of instruction on overall verbal fluency performance on the one hand and sex differences therein on the other hand.

The third set of analyses (see “Effect of instruction on verbal fluency strategies” section) addresses the effect of instruction on verbal fluency strategies and sex differences therein.

To control for multiple comparisons, Sidak post hoc corrections were used.

We first performed those analyses (sections “Neutral instructions,” “Effect of instruction and sex on overall verbal fluency performance (number of words produced)” and “Effect of instruction on verbal fluency strategies”) including both, phonemic and semantic, tasks in order to evaluate whether the type of task influenced the effects of sex or instruction on the number of words, cluster size, or number of switches. However, such a large statistical model may be underpowered with the current sample size to detect significant higher-order interactions. Furthermore, it has repeatedly been suggested that phonemic and semantic fluency tasks, while sharing some common verbal processing mechanisms, require different cognitive mechanisms and should therefore be viewed as different cognitive tasks (e.g., Henry and Crawford 2004; Milner and Petrides 1984; Troyer et al. 1997). Therefore, all analyses were also performed separately for the phonemic and semantic task to address effects of sex and instruction specific to each task.

Finally, a series of correlation analyses (see “Relationship between verbal fluency strategies and overall performance” section) was used to establish the relationship between strategies and overall performance under neutral conditions and when participants are influenced by specific instructions.

## Results

### Neutral instructions

#### Effects of sex and task on overall verbal fluency performance (number of words produced)

**Fig. 1:**
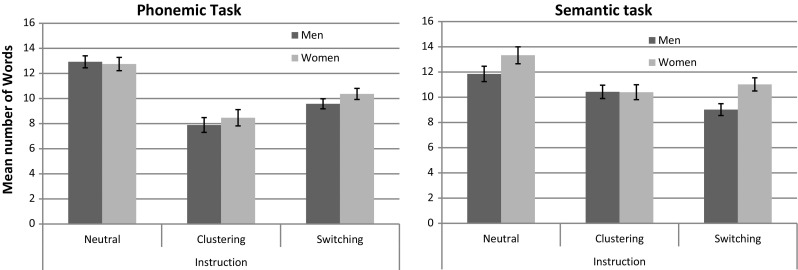
Mean number of words in the phonemic and semantic fluency task. Total number of words produced by men and women with different instructions in a phonemic and semantic verbal fluency task. *Error bars* represent standard errors of means

To test for sex differences in verbal fluency at baseline, a 2 × 2 ANOVA with the within-participants factor “*task*” (phonemic vs. semantic) and the between-participants factor “*sex*” was computed for the dependent variable “*number of words*” in the neutral instruction. Means are also displayed in Fig. 1. All analyses were performed using IBM SPSS, Statistics 22.

Neither the main effect of “*sex*” (*F*_(1,40)_ = 0.99, *p* = 0.33,* η*_*p*_^2^ = 0.03), nor the main effect of “*task*” were significant (*F*_(1,40)_ = 0.27, *p* = 0.61, *η*_*p*_^2^ = 0.01). Furthermore, the interaction between “*task*” and “*sex*” was non-significant, although of medium effect size (*F*_(1,40)_ = 2.93, *p* = 0.10, *η*_*p*_^2^ = 0.07). Thus, participants produced a comparable number of words in the phonemic and semantic task and men and women did not differ significantly in the number of words generated under neutral instructions. Task-specific analyses revealed that the number of words produced did not differ significantly between men and women in either the phonemic (*t*_(40)_ < 0.26, *p* = 0.80, *d* = 0.08) or the semantic task (*t*_(__4__0__)_ = −1.64, *p* = 0.11, *d* = 0.51).

#### Effects of sex and task on clustering and switching during verbal fluency

**Fig. 2:**
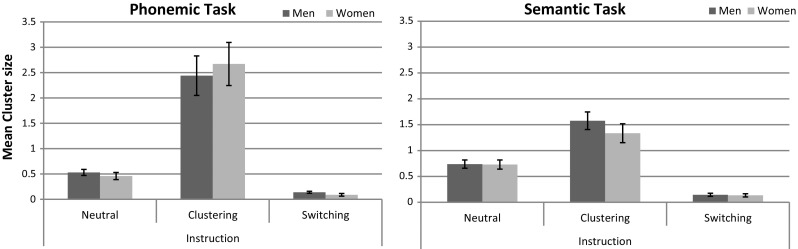
Average cluster size in the phonemic and semantic fluency task. Average cluster size in men and women with different instructions in a phonemic and semantic verbal fluency task. *Error bars* display standard errors of means

**Fig. 3:**
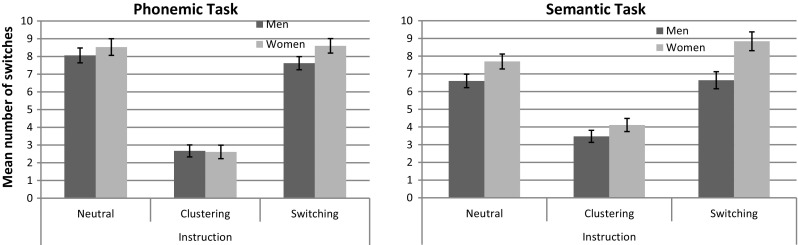
Mean number of switches in the phonemic and semantic fluency task. Mean number of switches produced by men and women with different instructions in a phonemic and semantic verbal fluency task. *Error bars* are based on standard errors of means

To test whether “*sex*” or “*task*” had a significant effect on strategy use during verbal fluency at baseline as observed by Weiss et al. (2006), we computed separate 2 × 2 ANOVAs on “*cluster size*” and “*number of switches*” for the neutral instruction. Results are displayed in Figs. 2 and 3, respectively.

##### *Cluster size*

The main effect of “*sex*” was non-significant (*F*_(1,40)_ = 0.18, *p* = 0.67, *η*_*p*_^2^ = 0.01), indicating that men and women did not differ in cluster size in the neutral condition. The main effect of “*task*” was significant (*F*_(1,40)_ = 13.16, *p* = 0.001 *η*_*p*_^2^ = 0.25), indicating that participants produced larger clusters in the semantic compared to the phonemic task. This effect was irrespective of sex as revealed by a small and non-significant “*sex*” × “*task*” interaction (*F*_(1,40)_ = 0.20, *p* = 0.66, *η*_*p*_^2^ = 0.01). Results are displayed in Fig. 2. Task-specific analyses confirmed that cluster size did not differ significantly between men and women in either the phonemic or the semantic task (both *t*s_(40)_ < .71, *p* > .48, *d* < .22).

##### *Number of switches*

The main effect of “*sex*” was non-significant, although of moderate effect size (*F*_(1,40)_ = 2.69, *p* = 0.11, *η*_*p*_^2^ = 0.06), indicating that men and women did not differ significantly in the number of switches. By contrast, a significant main effect of “*task*” (*F*_(1,40)_ = 9.82, *p* = 0.003, *η*_*p*_^2^ = 0.20) indicated that participants switched more often between categories in the phonemic task compared to the semantic task. The non-significant “*task*” × “*sex*” interaction revealed that the task influence was irrespective of sex in the neutral instruction condition (*F*_(1,40)_ = 0.75, *p* = 0.39, *η*_*p*_^2^ = 0.02). Task-specific analyses revealed a slightly higher number of switches in women compared to men (*t*_(40)_ = −1.93, *p* = 0.06, *d* = 0.60) in the semantic task, while men and women did not differ in the number of switches in the phonemic task (*t*_(40)_ = −0.75, *p* = 0.46, *d* = 0.23). All means are displayed in Fig. 3.

### Effect of instruction and sex on overall verbal fluency performance (number of words produced)

To test whether different instructions affected the number of words produced differently in men and women, a 2 × 3 × 2 mixed ANOVA was carried out on the dependent variable number of words with the within-participants factors “*task*” (phonemic vs. semantic) and “*instruction*” (neutral vs. clustering vs. switching) and the between-participants factor “*sex*” (see Fig. 1).

The main effect of “*sex*” was non-significant (*F*_(1,40)_ = 2.25, *p* = 0.14, *η*_*p*_^2^ = 0.05), indicating that, overall, there was no significant difference in the total number of words produced between men and women. A significant result was observed for the main effect of “*task*” (*F*_(1,40)_ = 8.60, *p* = 0.01, *η*_*p*_^2^ = 0.18). “*Task*” did however not interact with “*sex*” (*F*_(1,40)_ = 2.67, *p* = 0.11, *η*_*p*_^2^ = 0.06), indicating that participants produced overall more words in the semantic, compared to the phonemic task, irrespective of sex.

Furthermore, we observed a large and highly significant main effect of “*instruction*” (*F*_(2,80)_ = 66.78, *p* < 0.001, *η*_*p*_^2^ = 0.63). “*Instruction*” did not interact with “*sex*” (*F*_(2,80)_ = 1.65, *p* = 0.20, *η*_*p*_^2^ = 0.04) or “*sex*” × “*task*” (*F*_(2,80)_ = 1.69, *p* = 0.19, *η*_*p*_^2^ = 0.04), indicating that the difference in word generation between the two phonemic and semantic tasks and the different instruction conditions did not depend on sex. However, the significant main effects of “*task*” and “*instruction*” were qualified by a significant “*task*” × “*instruction*” interaction (*F*_(2,80)_ = 8.66, *p* < 0.001, *η*_*p*_^2^ = 0.18).

Separate analyses by task revealed that the main effect of “*instruction*” was significant in both tasks (both *F*_(2,80)_ > 13.89, both *p* < 0.001, both, *η*_*p*_^2^ > .26). In both tasks, the overall number of words produced was significantly lower in the clustering and switching instruction compared to the neutral instruction, although mean differences were larger in the phonemic task (neutral vs. clustering: MD = 4.65, SE = 0.41, *p* < 0.001; neutral vs. switching: MD = 2.86, SE = 0.27, *p* < 0.001) than in the semantic task (neutral vs. clustering: MD = 2.17, SE = 0.61, *p* = 0.003; neutral vs. switching: MD = 2.57, SE = 0.52, *p* < 0.001). However, the number of words was significantly higher in the switching compared to the clustering instruction only in the phonemic task (MD = 1.79, SE = 0.40, *p* < 0.001), but not in the semantic task (MD = −0.40, SE = 0.42, *p* = 0.73). This indicates a stronger effect of instruction for the phonemic compared to the semantic task.

The interaction between “*sex*” and “*instruction*” was non-significant in both tasks (both *F*_(2.80)_ < 2.00, both *p* > .14, *η*_*p*_^2^ < 0.05). However, a significant main effect of “*sex*” was observed only in the semantic task (*F*_(1,40)_ = 4.76, *p* = 0.04, *η*_*p*_^2^ = 0.11), but not in the phonemic task (*F*_(1,40)_ = 0.44, *p* = 0.51, *η*_*p*_^2^ = 0.01). Women produced more words compared to men in the semantic task, but not in the phonemic task. Since the difference was non-significant under neutral instructions, post hoc Sidak tests were performed separately for each instruction to evaluate which instruction showed the strongest difference. While no sex differences were observed under the clustering instruction (MD = 0.02, SE = 0.80, *p* = 0.98), a large sex difference was observed under the switching instruction (MD = 2.00, SE = 0.70, *p* = 0.007).

### Effect of instruction on verbal fluency strategies

To confirm stronger clustering during the clustering instruction and stronger switching during the switching instruction, two separate 3 (“*instruction*”) × 2 (“*task*”) × 2 (“*sex*”) ANOVAs were performed on “*cluster size*” and “*number of switches*.” Results are displayed in Figs. 2 and 3, respectively.

#### Effect of instruction on cluster size

As expected, there was a highly significant main effect of “*instruction*” (*F*_(2.80)_ = 114.39, *p* < 0.001, *η*_*p*_^2^ = 0.74). Like at baseline there were no significant main effects of “*sex*” (*F*_(1,40)_ = 0.04, *p* = 0.85, *η*_*p*_^2^ = 0.001) and no significant interaction between “*task*” and “*sex*” (*F*_(1,40)_ = 0.36, *p* = 0.55, *ƞ*_*p*_^2^ = 0.01). “*Instruction*” did not interact with “*sex*” and did not show a threefold interaction with “*task*” and “*sex*” (both *F*s_(2.80)_ < 0.70, *p* > .50, *ƞ*_*p*_^2^ < 0.02). Means indicated that cluster size was highest in the clustering condition compared to the other instruction conditions.

The main effect of “*task*” (*F*_(1,40)_ = 7.27, *p* = 0.01, *ƞ*_*p*_^2^ = 0.15) and the “*task*” × “*instruction*” interaction was however significant (*F*_(2.80)_ = 16.0, *p* < 0.001, *ƞ*_*p*_^2^ = 0.29). Means indicated that participants produced larger clusters in the phonemic compared to the semantic task.

Task-specific analyses revealed a highly significant main effect of “*instruction*” in both tasks (*F*_(2.80)_ > 64.71, *p* < 0.001, *ƞ*_*p*_^2^ > .62). In both tasks, the average cluster size was significantly lower in the neutral and switching instruction compared to the clustering instruction, although mean differences were larger in the phonemic task (clustering vs. neutral: MD = 2.06, SE = 0.28, *p* < 0.001; clustering vs. switching: MD = 2.44, SE = 0.29, *p* < 0.001) than in the semantic task (clustering vs. neutral: MD = 0.72, SE = 0.13, *p* < 0.001; clustering vs. switching: MD = 1.31, SE = 0.13, *p* < 0.001). Also in both tasks, the average cluster size was significantly higher in the neutral compared to the switching instruction (phonemic: MD = 0.38, SE = 0.05, *p* < 0.001, semantic: MD = 0.60, SE = 0.06, *p* < 0.001). This indicates a stronger effect of instruction for the phonemic compared to the semantic task.

The main effects of “*sex*” and the “*sex*”x“*instruction*” interactions were non-significant in both tasks (all *F*_(1,40)_ < 0.73, all *p* > .40, all *ƞ*_*p*_^2^ < 0.02).

#### Effects of instruction on the number of switches

A highly significant main effect of “*instruction*” was also observed on the “*number of switches*” (*F*_(2.80)_ = 235.27, *p* < 0.001, *ƞ*_*p*_^2^ = 0.86). The main effect of instruction was however qualified by significant “*instruction*” × “*task*” (*F*_(2.80)_ = 9.83, *p* < 0.001, *ƞ*_*p*_^2^ = 0.20) and “*instruction*” × “*sex*” (*F*_(2.80)_ = 3.58, *p* = 0.03, *ƞ*_*p*_^2^ = 0.08) interactions. While the main effect of “*task*” remained non-significant (*F*_(1,40)_ = 0.36, *p* = 0.55, *ƞ*_*p*_^2^ = 0.01), “*sex*” had a significant effect on the number of switches (*F*_(1,40)_ = 6.19, *p* = 0.02, *ƞ*_*p*_^2^ = 0.13), which was further qualified by a significant “*task*” × “*sex*” interaction (*F*_(1,40)_ = 4.41, *p* = 0.04, *ƞ*_*p*_^2^ = 0.10). The threefold interaction between “*task*,” “*instruction*” and “*sex*” was however non-significant (*F*_(2.80)_ = 0.20, *p* = 0.82, *ƞ*_*p*_^2^ = 0.01).

Task-specific analyses confirmed a large significant main effect of “*instruction*” in both tasks (both *F*s(2.80) > .60.60, *p*s < 0.001, *ƞ*_*p*_^2^ > .60). In both tasks, the number of switches did not differ significantly between neutral and switching instructions (all |MD| < 0.61, all SE > 0.32, all *p* > .26). Also, in both tasks and sexes, the number of switches was significantly lower in the clustering instruction compared to the neutral and the switching instruction. Mean differences were higher in the phonemic (both MD < −5.47, both SE < 0.34, both *p* < 0.001) compared to the semantic task (both MD < −3.36, both SE < 0.39, both *p* < 0.001), and for women (both MD < −4.75, both SE < 0.37, both *p* < 0.001) compared to men (both MD < −4.06, both SE < 0.33, both *p* < 0.001). This indicates a stronger effect of instruction for the phonemic compared to the semantic task and for women compared to men.

The main effect of “*sex*” was non-significant in the phonemic task (*F*_(1,40)_ = 1.21, *p* = 0.28, *ƞ*_*p*_^2^ = 0.03), but highly significant in the semantic task (*F*_(1,40)_ = 10.70, *p* = 0.002, *ƞ*_*p*_^2^ = 0.21). Women switched significantly more often between categories compared to men in the semantic fluency task. Sidak post hoc tests revealed a large sex difference in the number of switches in the switching instruction (MD = 1.59, SE = 0.49, *p* = 0.002), but not in the neutral and clustering instructions (both MD < 0.78, both SE > .39, both *p* > .10), although the “*sex*”x“*instruction*” was non-significant in both tasks (*F*s_(2.80)_ < 1.24, *p*s > 0.30, *ƞ*_*p*_^2^ < 0.03).

### Relationship between verbal fluency strategies and overall performance

To test whether verbal fluency strategies related to overall verbal fluency performance under neutral instructions as suggested by Weiss et al. (2006), the average cluster size and the average number of switches were correlated with the average number of words produced in the phonemic and semantic task in general and separately for men and women. The significance level was thus Bonferroni corrected to *p* < 0.001 since a total of 24 correlations were performed for the cluster size and number of switches, respectively (see below). All correlations are summarized in Tables 1 and 2. If we refer to the size of the correlation (e.g., “large positive correlation”), we interpreted those according to Cohen’s interpretation of effect sizes (Cohen 1988).

**Table 1:** Correlations between the average cluster size and overall number of words produced under each instruction in the phonemic and semantic fluency task

Cluster size	Neutral		Clustering		Switching	
	Phonemic	Semantic	Phonemic	Semantic	Phonemic	Semantic
Total	0.06	0.40	0.09	0.36~	0.09	0.06
Men	0.31	0.10	−0.01	0.11	0.15	0.13
Women	−0.17	0.73***	0.13	0.73***	0.12	0.04

*P* values were Bonferroni corrected to *p* < 0.001(***)

**Table 2:** Correlations between the number of switches and overall number of words produced under each instruction in the phonemic and semantic fluency task

Switches	Neutral		Clustering		Switching	
	Phonemic	Semantic	Phonemic	Semantic	Phonemic	Semantic
Total	0.74***	0.60***	0.53***	0.36	0.85***	0.88***
Men	0.65~	0.69***	0.30	0.59	0.82***	0.86***
Women	0.82***	0.36	0.68~	−0.06	0.86***	0.85***

*P* values were Bonferroni corrected to *p* < 0.001(***)

#### Cluster size

In the phonemic task, cluster size was not correlated with the overall number of words produced in either men or women in any instruction (all *r* < .31, all *p* > .15).

Also, in the semantic task, when considering the total sample, cluster size was not significantly correlated with the overall number of words produced in any instruction (all <.40, all *p* > 0.009). Separate analyses for men and women did however reveal a significant association between cluster size and overall performance under neutral and clustering instructions in women (both *r*_(18)_ > .73, both *p* < 0.001), but not in men (both *r*_(22)_ < .11, both *p* > .62). Fisher’s z comparisons revealed that the correlations between cluster size and overall performance (i.e., the number of words) did significantly differ between men and women in the neutral, as well as in the clustering condition (both *z* > 2.49 *p* < 0.006), indicating that in women the cluster size was more strongly correlated with overall performance than in men, at least under the neutral and the clustering instruction.

#### Number of switches

In both the phonemic and the semantic task, the number of switches was significantly positively correlated with the number of words produced in the total sample under neutral and clustering instructions (all *r* > 0.53, all *p* < 0.001). In the phonemic task, these correlations were confirmed for both the male and female subsample. In the semantic task under neutral instructions however, the number of switches was significantly correlated with the number of words produced in men (*r*_(22)_ = 0.69, *p* < 0.001), but not in women (*r*_(18)_ = 0.36, *p* = 0.13). A Fisher’s z test however indicated that these correlations did not differ significantly (*z* = −0.92, *p* = 0.18).

Under the clustering instruction, the association between number of words produced and number of switches was confirmed in the overall sample for the phonemic task *(r*_(41)_ = 0.53, *p* < 0.001), but not for the semantic task (*r*_(41)_ = 0.36, *p* = 0.02). In the phonemic task, the correlation was driven by the female subsample (*r*_(18)_ = 0.68, *p* = 0.001), while the association was by trend smaller in men (*r*_(22)_ = 0.30, *p* = 0.16; *z* = −1.55, *p* = 0.061). In the semantic task, a trend association was observed in men (*r*_(22)_ = 0.59, *p* = 0.003), which was significantly stronger than in women (*r*_(18)_ = −.06, *p* = 0.81; *z* = 2.20, *p* = 0.01).

## Discussion

The present study was designed to investigate whether performance in verbal tasks could be influenced by changing instructions, thereby influencing the cognitive strategies employed by men and women and causing changes in sex differences observed during the task. We chose a verbal fluency task to investigate this question, for which sex difference reports in performance and strategies in the literature are inconsistent. During neutral task conditions, it has been demonstrated that men tend to produce words clustered into phonemic or semantic subcategories, whereas women produce words by switching between categories (Lanting et al. 2009; Weiss et al. 2006).

We found that under the neutral instruction condition, when participants were asked to produce as many words as possible without restrictions, no significant sex differences could be observed, neither in the number of generated words, nor in the average size of phonemic or semantic clusters, nor for the number of switches.

After changing instructions to emphasize clustering or switching, respectively, the cluster size increased and the number of switches decreased in the clustering instruction, while under the switching instruction, participants still produced a comparable number of switches to the neutral instruction.

Nevertheless, under the clustering instruction we did not observe any sex differences in cluster size, number of switches, or the overall number of words produced. By contrast, under the switching instruction, sex differences were observed in the number of switches as well as the total number of words generated in the semantic fluency task. Under the switching instruction, women produced more switches and more words compared to men in the semantic fluency task.

Thus, significant sex differences in verbal fluency were observed only when participants were explicitly instructed to switch between categories, which increased the number of switches in women. One explanation for this could be that the switching instruction poses higher task demands than the neutral instruction by restricting the strategy. However, no sex differences were observed in the clustering instruction, which is of comparable task demands as the switching instruction. This suggests that sex differences are more likely to be detected in conditions that specifically require stronger switching between subcategories. Thus, different formulations of instructions could be one reason for the inconsistencies observed between previous studies on sex differences on verbal fluency. In particular, in the phonemic fluency task, the common instruction to avoid variants of the same word could emphasize switching and reduce clustering. This could explain why sex differences are more consistently observed in the phonemic task as opposed to the semantic task.

If sex differences in verbal fluency performance can be observed more strongly in conditions requiring switching, it is possible that they result from different roles of clustering and switching for overall performance in men and women. To investigate this assumption, the relationship between overall verbal fluency performance and clustering or switching was assessed separately for men and women in the present study. Over all participants, the number of switches, but not the average cluster size, was significantly related to the number of words produced in both tasks under most instructions. This observation is in line with previous studies, suggesting that switching is the more successful strategy during verbal fluency (Koren et al. 2005; Troyer et al. 1997; Weiss et al. 2006). However, these correlation patterns differed between men and women and between phonemic and semantic fluency tasks, indicating that strategies are of different importance for men and women in different tasks. Notably, correlation patterns aligned across sexes and tasks under the switching instruction when performance differences became apparent. This suggests that under the neutral and clustering instructions, men and women weighted the instructions differently.

Under the neutral and clustering instructions, the influence of switching on performance in the phonemic task seems to be driven by the female subsample, while in the semantic task this relationship is driven by the male subsample. By contrast, women even display an impact of clustering on performance in the semantic task. These results shed new light on the idea that switching is especially important for the phonemic task, while both switching and clustering are important for overall fluency performance in the semantic task, where associations between words are higher (Henry and Crawford 2004; Martin et al. 1994; Troyer et al. 1997). In fact, it seems that switching is important in the phonemic task only for women, while in the semantic task, switching is important for men, but clustering for women. If switching is indeed related to better performance in verbal fluency, this may explain previous observations that a female superiority in verbal fluency arises more consistently in the phonemic fluency task, whereas semantic fluency tasks sometimes even show a male superiority (Kempler et al. 1998).

The two tasks were also differentially influenced by the changes in instructions. After changing instructions, the cluster size increased more strongly under the clustering instruction in the phonemic task compared to the semantic task, but decreased more strongly under the switching instruction in the semantic task compared to the phonemic task. This is in line with previous research indicating stronger associations between words that are semantically related than between phonemically similar words (Martin et al. 1994; Martins et al. 2007; Troyer et al. 1997, Unsworth et al. 2010).

Accordingly, under the clustering instruction the number of words produced was higher in the semantic task as opposed to the phonemic task. Note that in the present study, the number of words produced did not differ between the phonemic and semantic task at baseline, even though the phonemic task has often been proposed to be more challenging (Gourovitch et al. 2000; Henry and Crawford 2004; Kavé et al. 2008; Milner and Petrides 1984; Mummery et al. 1996; Paulesu et al. 1997; Troyer et al. 1997; Unsworth et al. 2010).

A limitation of the present study is however that we did not control for other variables which have been observed to affect verbal fluency performance and strategies, like education and age (Brucki and Rocha 2004; Capitani et al. 1999; Sauzéon et al. 2010; Tombaugh et al. 1999; Troyer et al. 1997; Troyer 2000). Nevertheless, participants were balanced on education. All participants were students of the University of Salzburg. A quite narrow age range (18–29) was further used to reduce the possible confounding influence of age on sex differences in verbal fluency as well.

Furthermore, the written version of the verbal fluency task may not be comparable without limitation to the more commonly employed spoken versions. Most importantly, writing reduces the overall number of words produced in the given time frame and individual writing speed may present a confounding variable. On the other hand, the written version provides a more anonymous environment when performing the task. Participants may be more nervous and more constricted by fearing to say a wrong word.

Another limitation of the present study lies in the limited power to detect significant higher-order interactions with the current sample size.

In summary, the present results indicate that in a sample that shows no sex differences in semantic fluency performance under neutral instructions, sex differences in the number of switches and overall performance are observed with instructions requiring switching between categories. This effect is likely due to differential importance of clustering and switching for phonemic and semantic fluency between men and women. We therefore argue that it is of uttermost importance in sex difference research to phrase instructions in a neutral way and standardize instructions across studies. It is possible that variations between instructions used in different studies may have contributed to the current situation of inconsistent results regarding sex differences in verbal fluency performance.
